# Gas6/Axl signaling attenuates alveolar inflammation in ischemia-reperfusion-induced acute lung injury by up-regulating SOCS3-mediated pathway

**DOI:** 10.1371/journal.pone.0219788

**Published:** 2019-07-18

**Authors:** Chung-Kan Peng, Chin-Pyng Wu, Jr-Yu Lin, Shih-Chi Peng, Chien-Hsing Lee, Kun-Lun Huang, Chih-Hao Shen

**Affiliations:** 1 Division of Pulmonary and Critical Care Medicine, Department of Internal Medicine, Tri-Service General Hospital, National Defense Medical Center, Taipei, Taiwan; 2 Department of Critical Care Medicine, Landseed Hospital, Taoyuan, Taiwan; 3 Graduate Institute of Aerospace and Undersea Medicine, National Defense Medical Center, Taipei, Taiwan; 4 Department of Medical Research, Tri-Service General Hospital, National Defense Medical Center, Taipei, Taiwan; 5 Division of Endocrinology and Metabolism, Department of Internal Medicine, Tri-Service General Hospital, National Defense Medical Center, Taipei, Taiwan; 6 Graduate Institute of Medical Sciences, National Defense Medical Center, Taipei, Taiwan; Institut d'Investigacions Biomediques de Barcelona, SPAIN

## Abstract

**Background:**

Axl is a cell surface receptor tyrosine kinase, and activation of the Axl attenuates inflammation induced by various stimuli. Growth arrest-specific 6 (Gas6) has high affinity for Axl receptor. The role of Gas6/Axl signaling in ischemia-reperfusion-induced acute lung injury (IR-ALI) has not been explored previously. We hypothesized that Gas6/Axl signaling regulates IR-induced alveolar inflammation via a pathway mediated by suppressor of cytokine signaling 3 (SOCS3).

**Methods:**

IR-ALI was induced by producing 30 min of ischemia followed by 90 min of reperfusion in situ in an isolated and perfused rat lung model. The rats were randomly allotted to a control group and IR groups, which were treated with three different doses of Gas6. Mouse alveolar epithelium MLE-12 cells were cultured in control and hypoxia-reoxygenation (HR) conditions with or without Gas6 and Axl inhibitor R428 pretreatment.

**Results:**

We found that Gas6 attenuated IR-induced lung edema, the production of proinflammatory cytokines in perfusates, and the severity of ALI ex vivo. IR down-regulated SOCS3 expression and up-regulated NF-κB, and Gas6 restored this process. In the model of MLE-12 cells with HR, Gas6 suppressed the activation of TRAF6 and NF-κB by up-regulating SOCS3. Axl expression of alveolar epithelium was suppressed in IR-ALI but Gas6 restored phosphorylation of Axl. The anti-inflammatory effect of Gas6 was antagonized by R428, which highlighted that phosphorylation of Axl mediated the protective role of Gas6 in IR-ALI.

**Conclusions:**

Gas6 up-regulates phosphorylation of Axl on alveolar epithelium in IR-ALI. The Gas6/Axl signaling activates the SOCS3-mediated pathway and attenuates IR-related inflammation and injury.

## Introduction

Acute lung injury (ALI) induced by ischemia-reperfusion (IR) manifests as nonspecific alveolar damage, lung edema, and hypoxemia within hours of the blood supply returning to ischemic lung tissue [1, 2]. Despite advancements in organ preservation and peri-operative care, IR-ALI remains a major cause of primary graft dysfunction after lung transplantation and respiratory insufficiency after cardiopulmonary bypass [3–6]. The rapid generation of reactive oxygen species (ROS) after reperfusion leads to a wide array of mediators produced from injured lung tissue, which activate the innate immune response and result in alveolar inflammation. The biochemical, cellular, and molecular alterations impair the integrity of the alveolar–capillary barrier, which causes IR-ALI [7–9].

The TAM family of kinases, including Axl, Tyro3, and MerTK, are cell-surface transmembrane receptors with regulated tyrosine kinase activity within their cytoplasmic domains [10–13]. Growth arrest-specific 6 (Gas6) is a secreted vitamin K-dependent ligand that binds to all three TAM receptors and has the highest affinity for Axl receptor [14–16]. Gas6/Axl signaling modulated autoimmune disorders in a mouse model [14, 17]. Gas6 administration decreased inflammation in mice with immune-mediated arthritis [18]. Axl interacts with a multi-domain intracellular protein involved in other signaling pathways, such as phosphatidylinositol 3-kinase (PI3K), phospholipase C (PLC), growth factor receptor-bound protein 2 (Grb2), C1 domain-containing protein (C1-TEN), NCK adaptor protein 2 (Nck2), Ran binding protein in microtubule organizing center (RanBPM), and suppressor of cytokine signaling (SOCS) [19–21]. Among the SOCS family, the best-studied members are SOCS1 and SOCS3. SOCS3 plays an important role as an intracellular negative regulator of inflammation [22].

Gas6/Axl signaling also modulated acute inflammation. In a study of dendritic cells exposed to lipopolysaccharide treatment, Axl signaling contributed to a self-regulating cycle of inflammation [16]. Another study showed that Gas6 administration inhibited the nuclear factor κB (NF-κB) pathway and secretions of tumor necrosis factor-α (TNF-α) and interleukin 6 (IL-6) by monocytes/macrophages after lipopolysaccharide (LPS) stimulation [23]. A clinical study has found that high concentrations of plasma Gas6 reflects microcirculatory abnormalities and the phagocytosis of dying cells in severe acute pancreatitis [24]. Plasma concentrations of Gas6 have also been correlated with disease severity in patients with severe sepsis and septic shock [25, 26].

Axl signaling is responsible for various responses, depending on the stimuli, cell types, and binding partners [14]. In a mouse study of ALI induced by cecal ligation and puncture, Gas6 inhibited neutrophil migration into the lung parenchyma. Simultaneously, Gas6 attenuated the inflammatory cascade and severity of lung injury, which improved the 10-day survival rate [27]. Axl is expressed throughout all cell types in adult tissues, not only the innate immune system [28]. In a mouse model of lung injury induced by high tidal volume, overdistention of lung endothelial cells activated ion channels and inactivated Axl [29]. Whether Gas6 protects the alveolar epithelium from acute inflammation remains uncertain.

The role of Gas6/Axl signaling in IR-ALI has not been explored previously. The present study examined the protective effect of Gas6 in IR-ALI by an in situ isolated and perfused rat lung model. An in vitro hypoxia-reoxygenation (HR) model was designed to determine the mechanism through which Gas6/Axl signaling regulates IR-induced inflammation in the alveolar epithelium.

## Methods

### Isolated perfused lung model in rats

The experiments were conducted using Sprague-Dawley rats (350 ± 20 g), which were purchased from BioLASCO Taiwan Co., Ltd. (Taipei, Taiwan) with 2 rats per cage. The animals were cared for according to the guidelines of the National Institutes of Health (US NIH). The Institutional Animal Care and Use Committee of the National Defense Medical Center approved the experiments. Previously described methods were used to prepare the isolated and perfused rat lungs. Briefly, intraperitoneal sodium pentobarbital was used to anesthetize the rats (50 mg kg^-1^), and tracheostomy was carried out. The rats were then ventilated with humidified air containing 5% CO_2_ using a positive end-expiratory pressure of 1 cm H_2_O. The ventilator settings also included a tidal volume of 3 mL and a frequency of 60 breaths/min.

A median sternotomy was the conducted, followed by the injection of heparin (1 U/g of BW) into the right ventricle. Next, 10 mL of blood was collected from the cardiac puncture. The isolated lung was perfused using 10 ml of collected blood and 10 mL of physiological salt solution, which contained 4% bovine serum albumin. An afferent cannula was inserted into the pulmonary artery, while an effluent cannula was inserted into the left atrium through the left ventricle to collect the effluent perfusate. The left atrial pressure represents the pulmonary venous pressure (PVP) and was monitored from a side arm of the outflow cannula. The pulmonary arterial pressure (PAP) was monitored in the side arm of the inflow cannula. A constant flow rate of 7 mL/min was provided using a roller pump. The changes in lung weight were recorded in real time by placing the isolated perfused lung in situ on an electronic balance.

### Experimental protocols

We used rat recombinant Gas6 (MBS1354209, Mybiosource, San Diego, CA, USA), a full length and gamma-carboxyglutamic acid-containing protein, in IR. The recombinant Gas6 protein was diluted by PBS solution to 25, 50, and 100 μg/ml. At the time of reperfusion, three doses (1.25, 2.5, or 5 μg/rat) of recombinant Gas6 protein or an equal volume of the vehicle (0.05 ml PBS solution) were administered via the perfusate (n = 6 per group by simple randomization). In the IR group, the lungs were subjected to ischemia by stopping ventilation and perfusion for 30 min, which was followed by reperfusion and ventilation for 90 min after the ischemia.

### MLE-12 cell culture

The mouse lung epithelial cell line MLE-12 (CRL-2110) was obtained from the American Type Culture Collection (ATCC, Manassas, VA, USA). The cells were cultured in DMEM/F-12 media (Biological Industries, Beth Haemek, Israel), which contained 2% fetal bovine serum (FBS; Invitrogen, Carlsbad, CA, USA) and other relevant supplements: 5 μg/ml of insulin, 10 μg/ml of transferrin, 2 mM of L-glutamine, 10 mM of HEPES (Biological Industries, Beth Haemek, Israel), 30 nM of sodium selenite, 10 nM of hydrocortisone, and 10 nM of β-estradiol (Sigma-Aldrich, St. Louis, MO, USA). The culture was carried out at 37°C in a humidified atmosphere that contained 5% CO_2_. The cells were passaged at approximately 80% confluence by incubation with 0.25% trypsin-EDTA. For the experiments, the cells were seeded in a six-well plate at a density of 3.5 x 10^4^ cells/cm^2^. They were then cultured for 24 h under the same conditions for expansion.

### Hypoxia-reoxygenation model in MLE-12 cells

After 24 h of incubation, the cells were pretreated with vehicle and 5, 10, or 20 ng/ml of mouse recombinant Gas6 protein (986-GS/CF, R&D Systems, Minneapolis, MN, USA). After 2 h of pretreatment, the cells were subjected to 24 h of hypoxia (5% CO_2_, 1% O_2_, and 94% N_2_), followed by reoxygenation for 1 h (5% CO_2_ in ambient air) at 37°C using a CO_2_/Tri-Gas Incubator (ASTEL). The Axl inhibitor R428 (100 nM) (Enzo Life Sciences, Inc., San Diego, CA, USA) was pretreated 2 h before Gas6 treatment.

### Bronchoalveolar lavage fluid and perfusate protein concentration and cytokine levels

Bronchoalveolar lavage fluid (BALF) was obtained at the end of the experiment. This was accomplished by lavaging the left lung twice with 2.5 mL of saline. Next, the fluid was centrifuged immediately at 200×g for 10 min to remove all cells and cellular debris. The protein levels were measured using a Pierce^TM^ BCA protein assay kit (Thermo Fisher Scientific). Commercially available ELISA kits (R&D Systems Inc., Minneapolis, MN, USA) were used to measure the levels of TNF-α and IL-6 in the BALF and cytokine-induced neutrophil chemoattractant 1 (CINC-1), IL-1β, IL-6, and TNF-α in the perfusate. Gas6 levels in the perfusate were quantified using an enzyme-linked immunosorbent assay kit (MyBioSource lab. Inc., San Diego, CA).

### Immunoblotting

Sodium dodecyl sulfate–polyacrylamide gel electrophoresis (SDS-PAGE) was used to separate the lysates (30 μg/lane) of lung and cell culture protein, which were then transferred to a polyvinylidene fluoride membrane (Millipore). The blots were incubated overnight with primary antibodies, including anti-Axl, anti- phosphorylated-Axl (1:1000, Bioss Inc., Beijing, China), anti-TNF receptor associated factor 6 (anti-TRAF6) (1:200, Santa Cruz Biotechnology, USA), anti-SOCS3, anti-phosphorylated-NF-κB p65, anti-IκB-α, anti-TATA (1:1000, Cell Signaling Technology, USA), and anti-β-actin (1:10000, Sigma Chemical Company, USA).

### Histopathology

Next, analyses were done to investigate the numbers of polymorphonuclear neutrophils, as well as the lung injury score in the lung tissue. Briefly, the lung tissues were fixed, sectioned, and stained with eosin and hematoxylin. Light microscopy was used to examine the morphology. The neutrophil infiltration in the airspace or vessel wall was examined using a minimum of 10 randomly selected fields. The thickening of the alveolar wall was also investigated. A four-point scale was used to score the lung damage as none (0), mild (1), moderate (2), or severe (3). Two pathologists who were blinded to the experimental conditions performed the scoring. The final lung injury score was obtained by summing the two resulting scores of the individual pathologists.

### Immunofluorescence staining

SOCS3 antibody (Santa Cruz Biotechnology, USA) was used for immunofluorescent labeling overnight at 4°C and then incubated for 1 h at room temperature using chicken anti-rabbit IgG-fluoresce in isothiocyanate (Santa Cruz Biotechnology, USA) as the secondary antibody. After washing, the slides were mounted using VECTASHIELD Antifade Mounting Medium with DAPI. A DeltaVision system (Applied Precision) was used to obtain images. The system comprises a wide-field inverted microscope (model IX-71; Olympus) with ×60/1.42 Plan Apo N or ×100/1.40 Super-Plan APO objectives. A CCD camera (Coolsnap HQ2; Photometrics) and Softworx analysis software (GE Healthcare) were used to capture the images.

### Quantitative real-time PCR

An RNA-spin total RNA extraction kit (Intron Biotechnology, Korea) was used to isolate the total RNA according to the manufacturer’s instructions. The synthesis of cDNA was performed using 2 μg of RNA and a High-Capacity cDNA Archive Kit (Applied Biosystems, Foster City, CA, USA). TaqMan assays (Applied Biosystems, Foster City, CA, USA) were used to perform quantitative real-time PCR for Axl, Gas6, SOCS3 and GAPDH. Each sample was analyzed in triplicate on a 96-well plate. The plate was briefly centrifuged and placed in a ViiA 7 qRT-PCR machine (Thermo Fisher Scientific, Waltham, MA, USA), and the analysis was performed using the following program: 2 min at 50°C, 10 min at 95°C, and 40 cycles of 15 s at 95°C and 1 min at 60°C. The 2-ΔΔCT method was used to calculate the relative gene expression.

### Statistical analysis

The data are expressed as the mean ± the standard deviation (SD). Groups were compared using one-way or two-way repeated-measures analysis of variance (ANOVA). Next, the Newman-Keuls test was used to perform a post hoc comparison. Statistical significance was defined using p < 0.05.

## Results

### Gas6 attenuates IR-induced lung edema ex vivo

**Fig 1:**
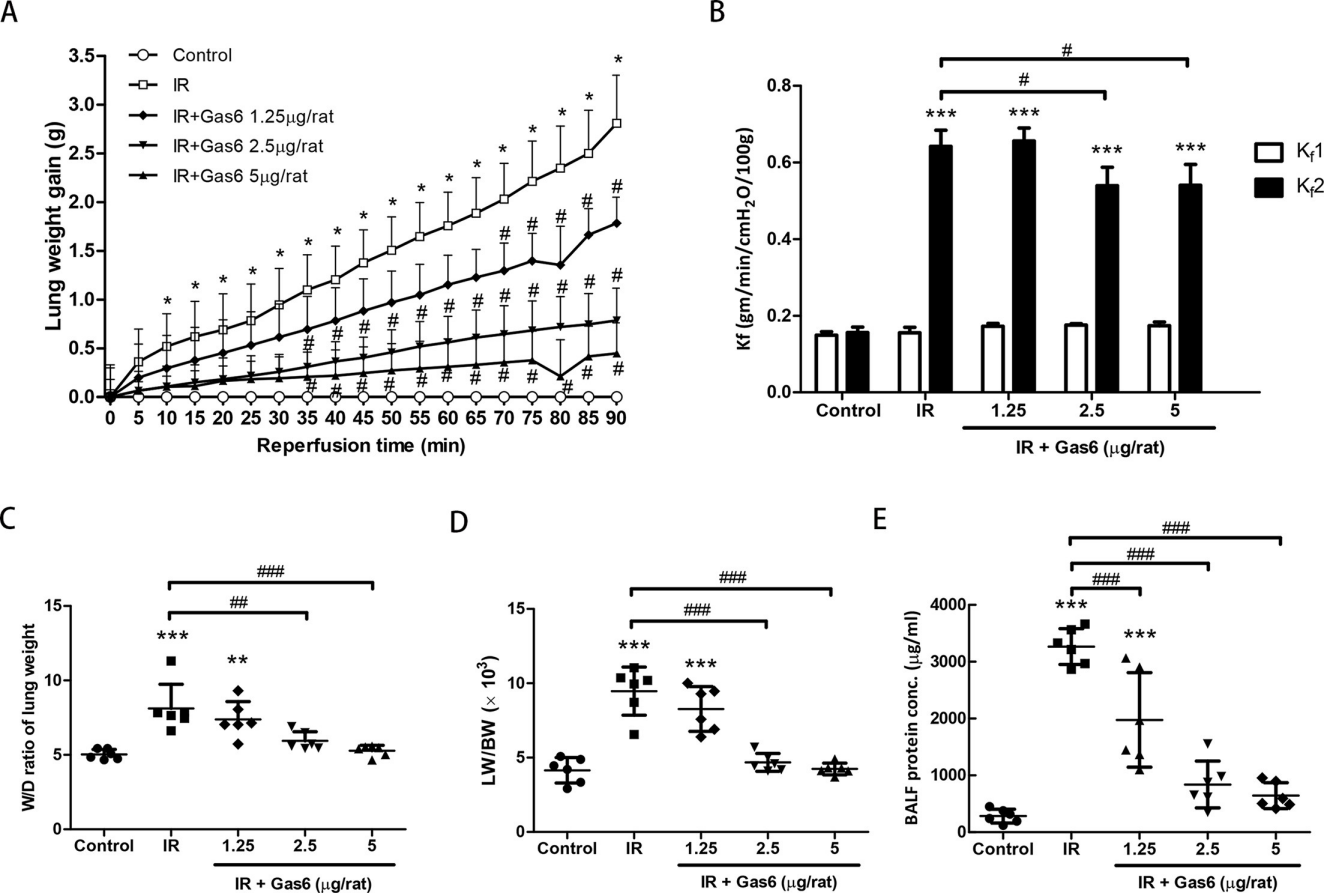
Effects of Gas6 on lung edema. (A) Lung weight gain, (B) pulmonary microvascular permeability (Kf), (C) ratios of lung wet/dry (W/D) weight, (D) lung weight/body weight (LW/BW), and (E) protein concentration in bronchoalveolar lavage fluid (BALF) in IR-ALI. The increase of these parameters in the IR group was significantly attenuated by pretreatment with Gas6. IR: ischemia–reperfusion. Data are expressed as the mean ± SD (n = 6 per group). * P < 0.05 compared with the control group; ** P < 0.01 compared with the control group; *** P < 0.001 compared with the control group; # P < 0.05 compared with the IR group; ## P < 0.01 compared with the IR group; ### P < 0.001 compared with the IR group.

IR increased the lung weight gain, vascular filtration coefficient (Kf), the ratio of lung weight to body weight (LW/BW), and the ratio of wet weight to dry weight ratio (W/D). The administration of 2.5 μg and 5 μg of Gas6 **s**ignificantly attenuated the increases of these parameters (Fig 1A–1D). These results suggest that Gas6 decreases lung edema in IR-ALI ex vivo. The protein concentration in BALF was measured as an indicator of dysfunction in the alveolar–capillary barrier, and the administration of 1.25, 2.5, and 5 μg of Gas6 significantly decreased the elevated protein concentration in the IR group (Fig 1E).

### Gas6 suppresses the production of proinflammatory cytokines in perfusates in IR-ALI

**Fig 2:**
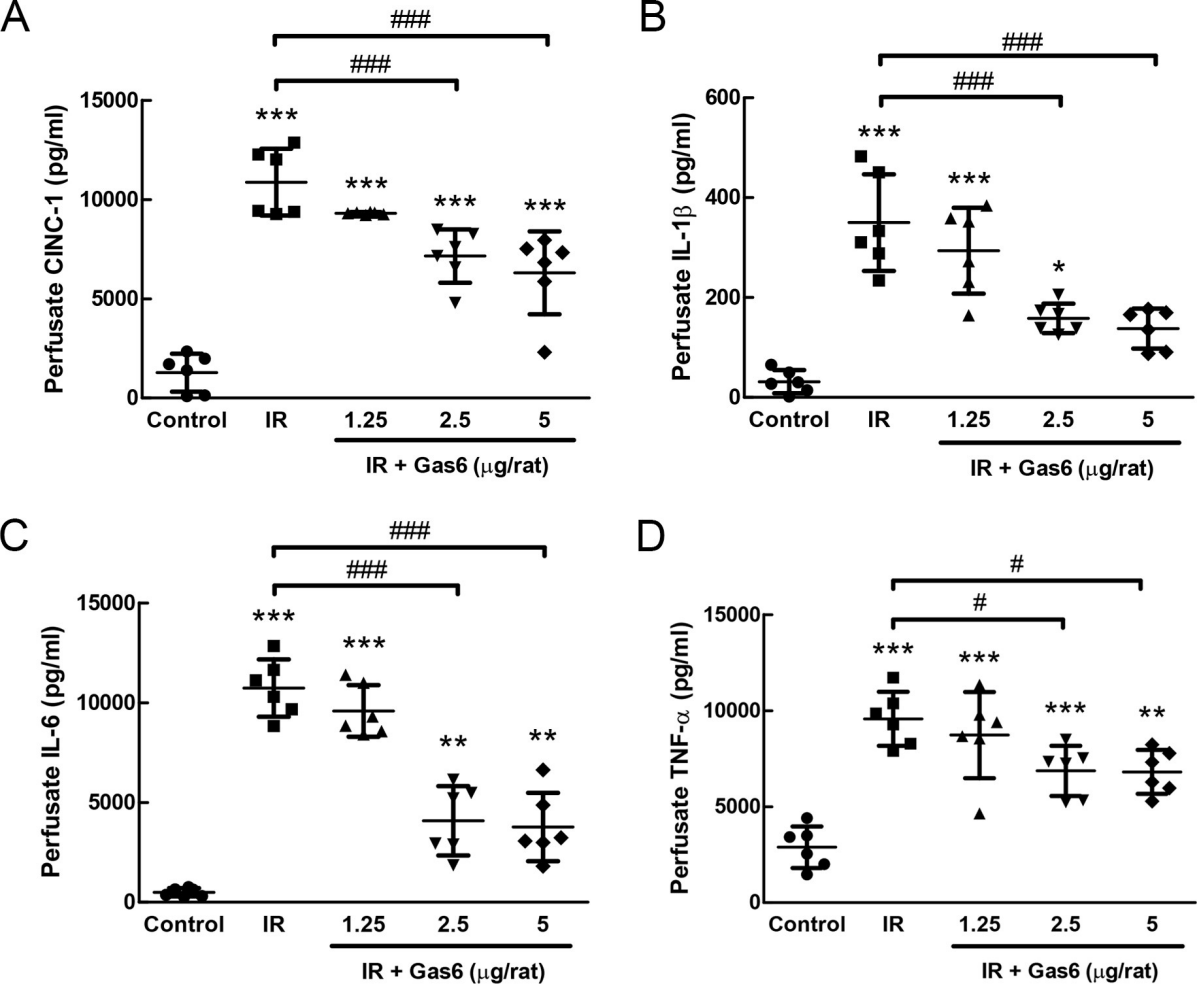
Effects of Gas6 on proinflammatory cytokines. (A) CINC-1, (B) IL-1β, (C) IL-6, and (D) TNF-α in IR-ALI. Gas6 attenuated the production of proinflammatory cytokines induced by IR. IR: ischemia–reperfusion. Data are expressed as the means ± SD (n = 6 per group). * P < 0.05 compared with the control group; ** P < 0.01 compared with the control group; *** P < 0.001 compared with the control group; # P < 0.05 compared with the IR group; ### P < 0.001 compared with the IR group.

Compared with those in the control group, IR significantly increased the perfusate levels of proinflammatory cytokines, such as CINC-1, IL-1β, IL-6, and TNF-α. The administration of 2.5 and 5 μg of Gas6 significantly attenuated these increases (Fig 2A–2D). These results suggest that Gas6 suppresses the local production of proinflammatory cytokines in IR-ALI.

### Gas6 attenuates the severity of IR-ALI

**Fig 3:**
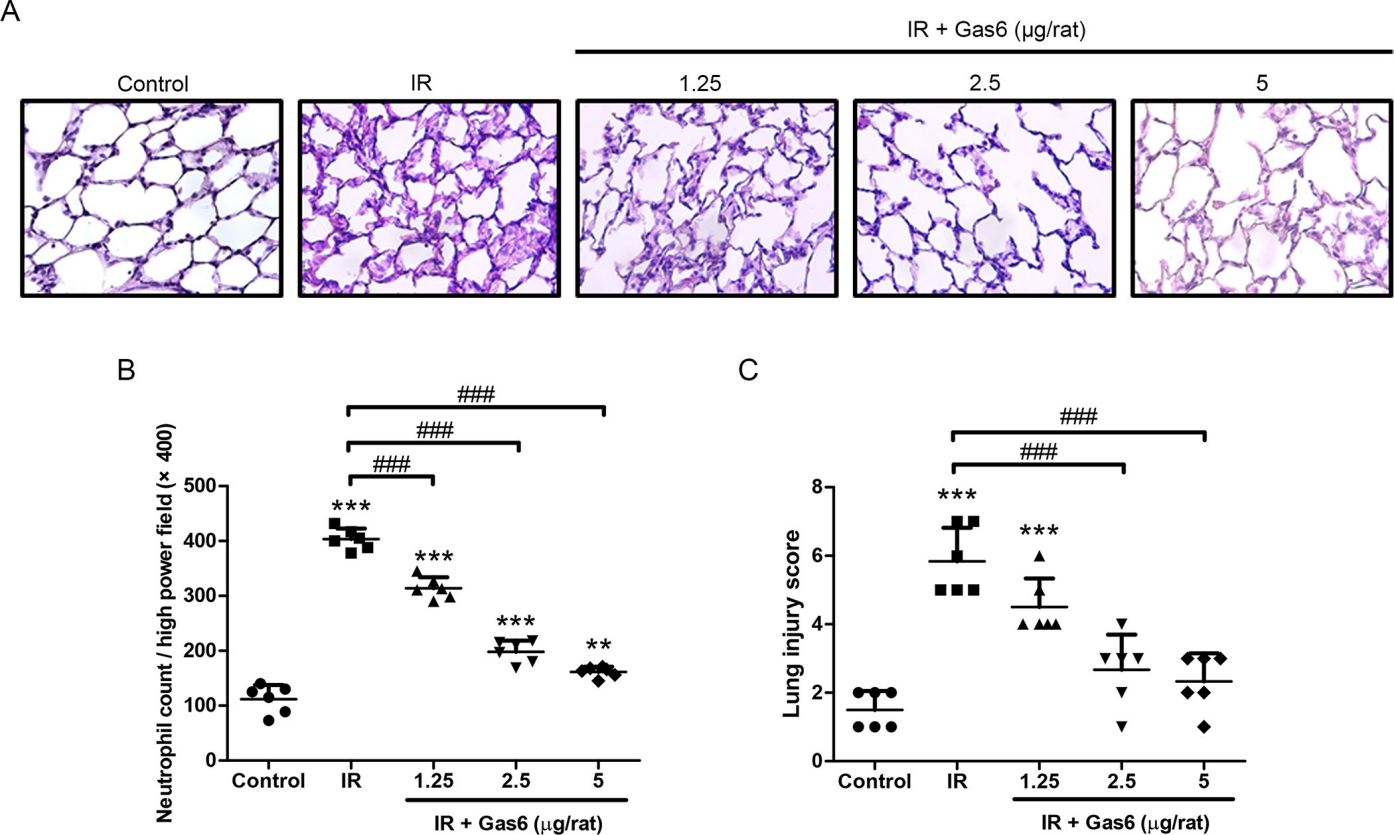
Effects of Gas6 on lung tissues. (A) Hematoxylin and eosin staining for lung tissue (200× magnification), (B) neutrophil count, and (C) lung injury score in IR-ALI. Gas6 attenuates the severity of IR-ALI. IR: ischemia–reperfusion. Data are expressed as the mean ± SD (n = 6 per group). ** P < 0.01 compared with the control group; *** P < 0.001 compared with the control group; ### P < 0.001 compared with the IR group.

Histological evaluation of lung tissues indicated a high neutrophil count and lung injury score after IR (Fig 3A–3C). The administration of 1.25, 2.5, and 5 μg of Gas6 significantly attenuated the increases in neutrophil count (Fig 3B). Administration of 2.5 and 5 μg of Gas6 significantly lessened the increases in lung injury score (Fig 3C). These results show that Gas6 attenuates the severity of IR-ALI.

### Gas6 modulates NF-κB in IR-ALI

**Fig 4:**
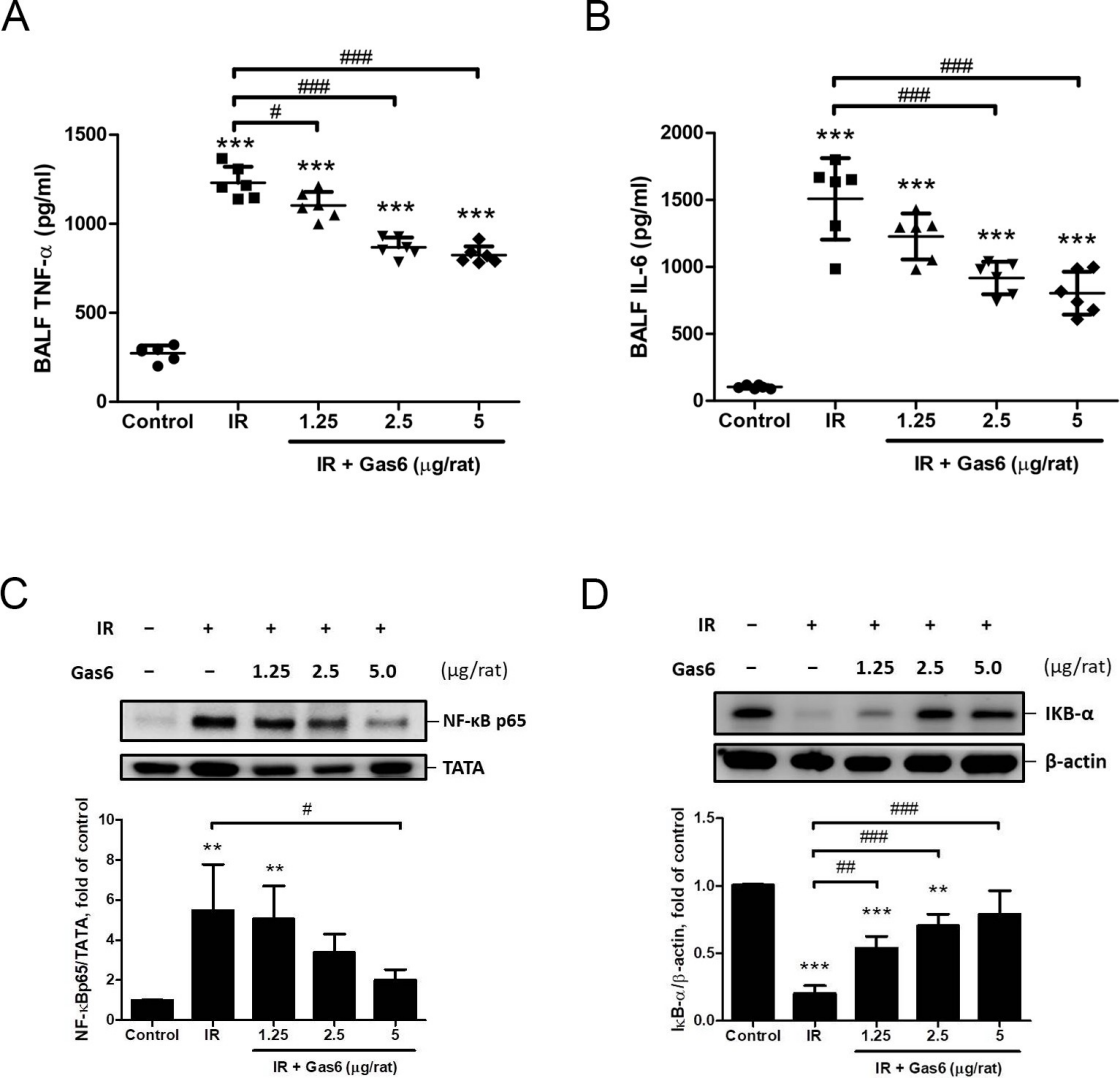
Effects of Gas6 on NF-κB of lung tissues. (A) Bronchoalveolar lavage fluid (BALF) TNF-α, (B) BALF IL-6, (C) NF-κB p65 levels of lung tissues, and (D) cytoplasmic IκB-α levels of lung tissues in IR-ALI. Gas6 modulates TNF-α and IL-6 levels in BALF and NF-κB expressions of lung tissues in IR-ALI. IR: ischemia–reperfusion. Data are expressed as the means ± SD (n = 6 per group for BALF and N = 4 per group for lung tissue). ** P < 0.01 compared with the control group; *** P < 0.001 compared with the control group; # P < 0.05 compared with the IR group; ## P < 0.01 compared with the IR group; ### P < 0.001 compared with the IR group.

Compared with the control group, IR significantly increased the TNF-α and IL-6 levels in BALF compared with those in the control group (Fig 4A and 4B). Western blot analysis indicated higher levels of nuclear NF-κB p65 in the IR group, whereas the level of cytoplasmic IκB-α was significantly suppressed (Fig 4C and 4D). The administration of 1.25, 2.5, and 5 μg of Gas6 significantly attenuated the increases in BALF TNF-α levels and restored the suppressed cytoplasmic IκB-α levels (Fig 4A and 4D). The administration of 2.5 and 5 μg of Gas6 significantly attenuated the increases in BALF IL-6 levels (Fig 4B). The administration of 5 μg of Gas6 significantly reduced the nuclear NF-κB p65 level in IR-ALI (Fig 4C). These results suggest that Gas6 modulates NF-κB in lung tissue in IR-ALI.

### Gas6 restores SOCS3 expression of alveolar epithelium in IR-ALI

**Fig 5:**
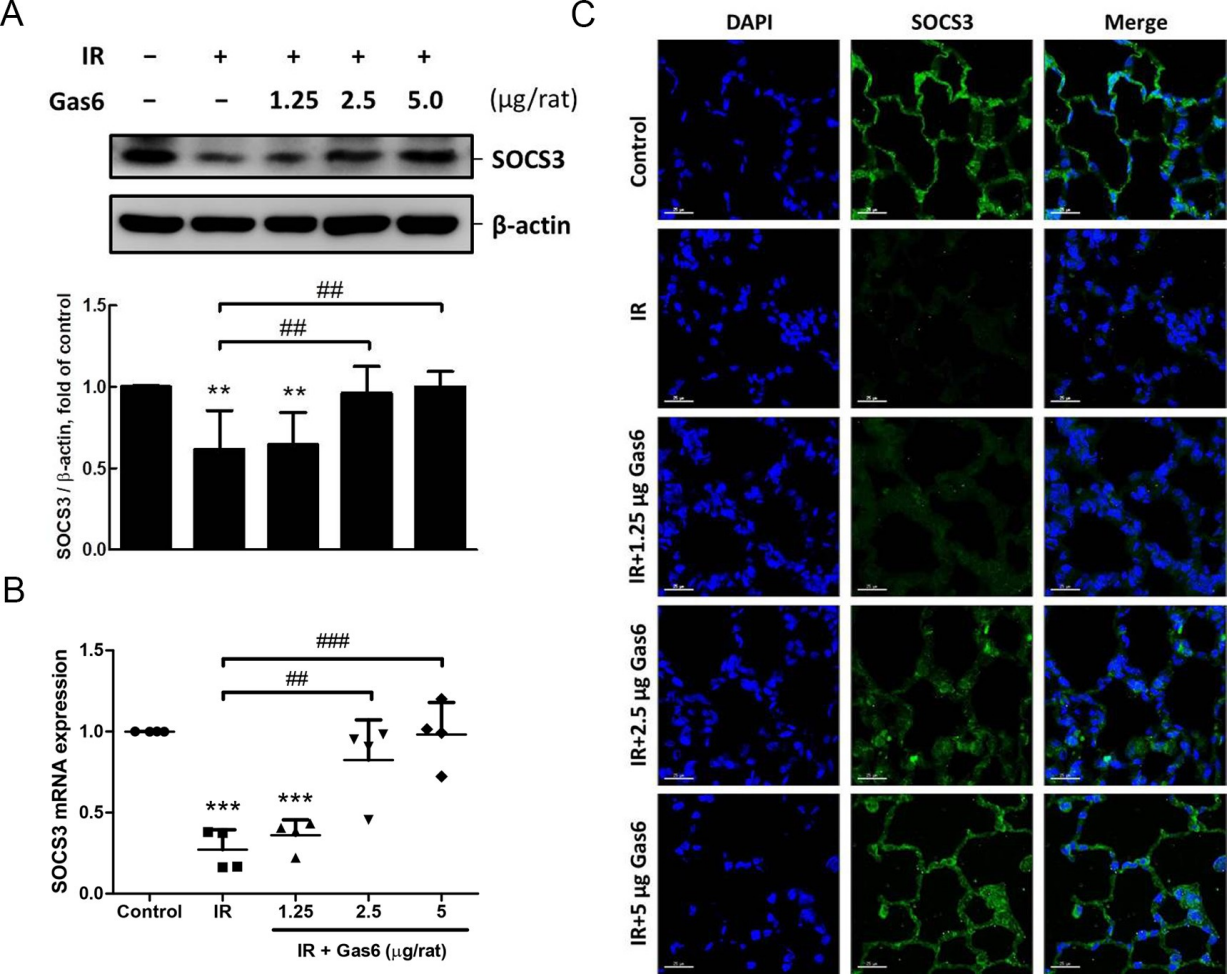
SOCS3 expression of lung tissues and alveolar epithelium. (A) SOCS3 protein levels in lung tissues determined by western blot analysis. (B) SOCS3 mRNA expressions in lung tissues. (C) Representative images of SOCS3 immunofluorescence staining (FITC-labeled green; original magnification ×400) of rat lung. Nuclei were counterstained with DAPI (blue). SOCS3 expressions of alveolar epithelium are significantly decreased after IR and restored by Gas6 treatment. IR: ischemia–reperfusion. Data are expressed as the mean ± SD (n = 4 per group). ** P < 0.01 compared with the control group; *** P < 0.001 compared with the control group; ## P < 0.01 compared with the IR group; ### P < 0.001 compared with the IR group.

IR significantly decreased the protein levels and mRNA expressions of SOCS3 in lung tissues. In comparison to the IR group, the administration of 2.5 and 5 μg of Gas6 restored SOCS3 protein levels and mRNA expressions (Fig 5A and 5B). The immunofluorescence results also showed that the expression of SOCS3 in alveolar epithelium was significantly lower in the IR group than in the control group, which was significantly increased by treatment with 2.5 and 5 μg of Gas6 (Fig 5C). These results suggest that Gas6 increases SOCS3 expression in IR-ALI and highlight its potential effect on the alveolar epithelium.

### Gas6 up-regulates the SOCS3-mediated pathway in alveolar epithelium with HR

**Fig 6:**
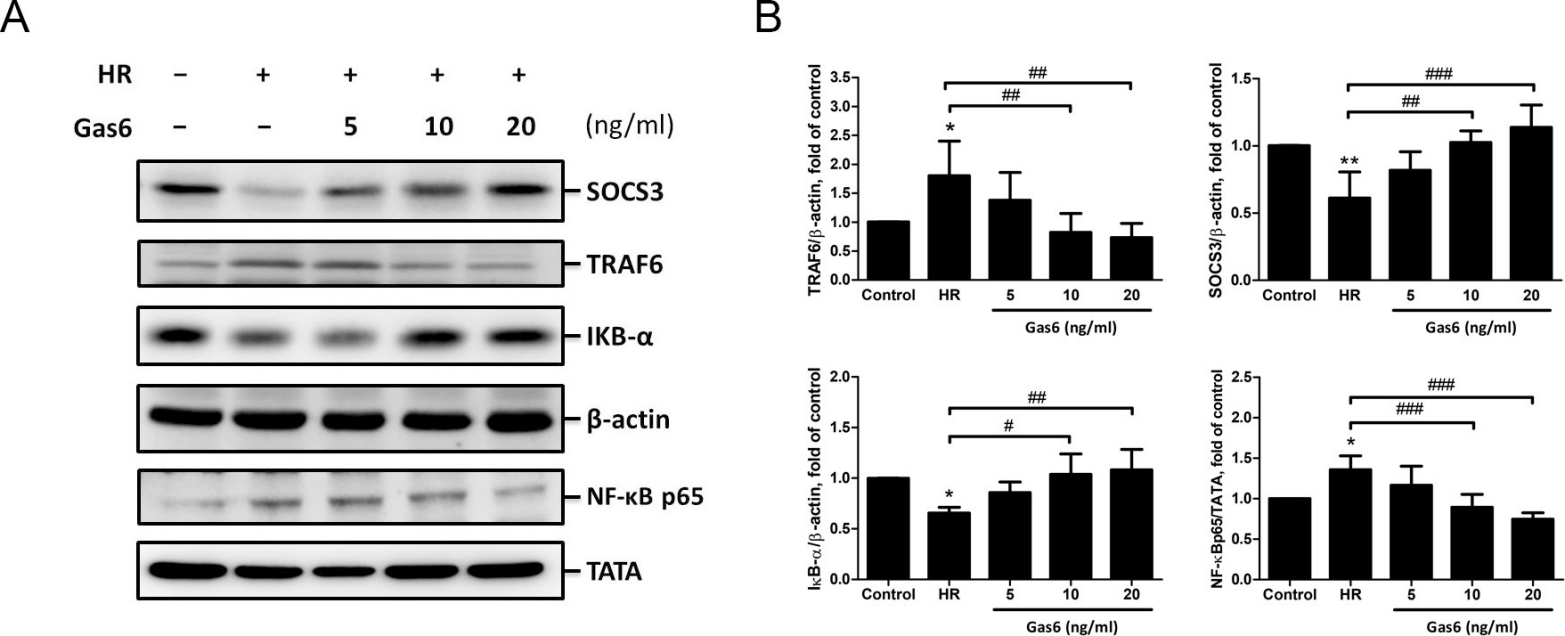
The SOCS3-mediated pathway in MLE-12 cells with HR. (A-B) SOCS3, TRAF6, cytoplasmic IκB-α, and nuclear NF-κB p65 levels determined by western blot analysis. TATA and β-actin served as loading controls for nuclear and cytoplasmic proteins, respectively. Gas6 up-regulates SOCS3 and down-regulates TRAF6 and NF-κB in HR model. HR: hypoxia-reoxygenation. Data are expressed as the means ± SD (n = 4 per group). * P < 0.05 compared with the control group; ** P < 0.01 compared with the control group; # P < 0.05 compared with the HR group; ## P < 0.01 compared with the HR group; ### P < 0.001 compared with the HR group.

To determine whether Gas6 modulates inflammation of alveolar epithelium in IR-ALI, SOCS3, TRAF6, and NF-κB were measured in MLE-12 cells with HR. The level of SOCS3 was suppressed by HR. In contrast, the NF-κB and its upstream TRAF6 were activated by HR. Pretreatment with 10 and 20 ng of Gas6 up-regulated SOCS3 and down-regulated the TRAF6/NF-κB pathway (Fig 6A and 6B). These results strengthen the idea that Gas6 directly acts on the alveolar epithelium, which restores SOCS3 expression and suppresses NF-κB in IR-ALI.

### Gas6 modulates the SOCS3 mediated pathway via up-regulating phosphorylated Axl in alveolar epithelium with HR

**Fig 7:**
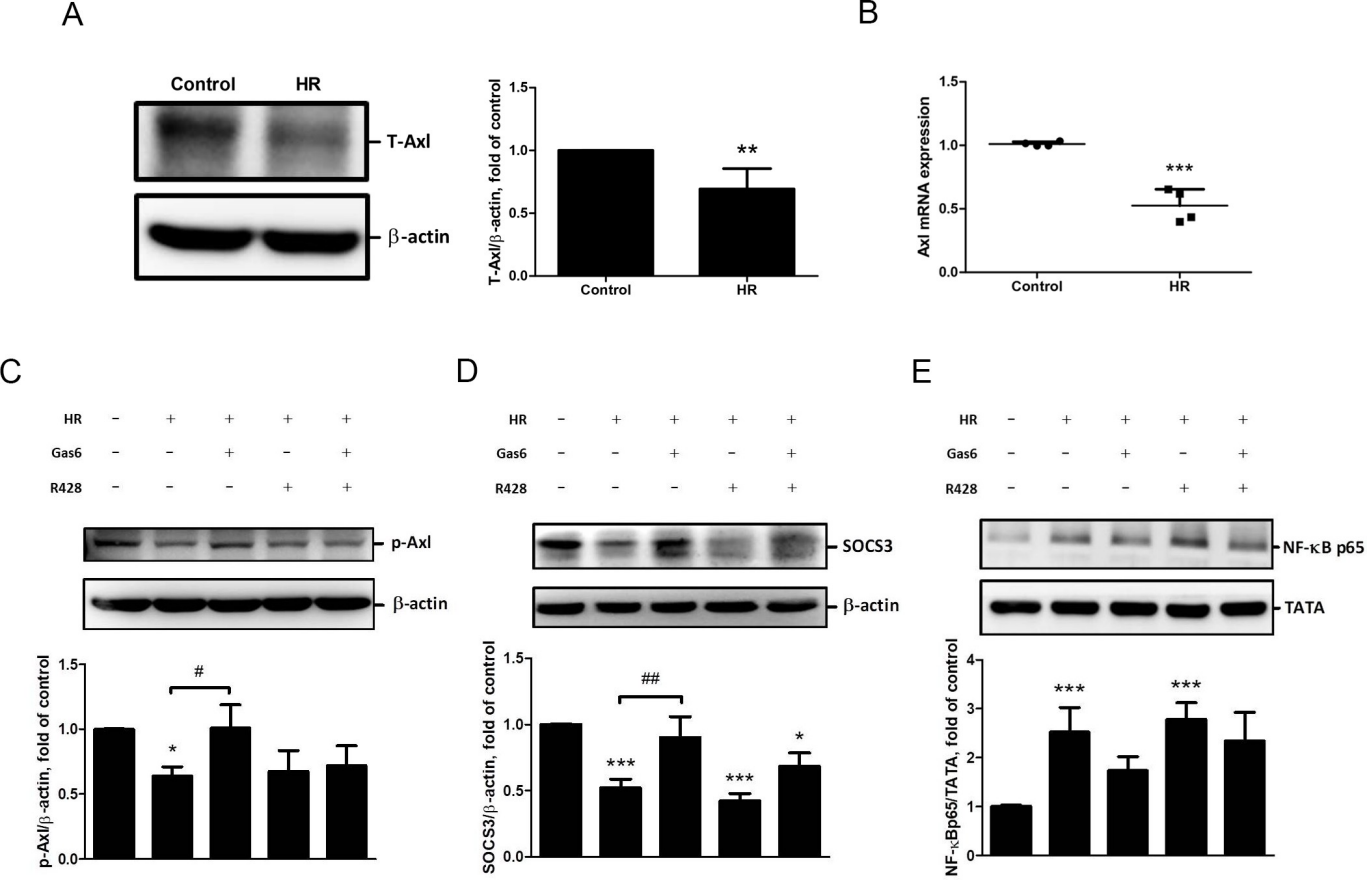
Modulation of Axl in MLE-12 cells with HR. (A) T-Axl protein levels determined by western blot analysis. (B) Axl mRNA expressions. (C) p-Axl, (D) SOCS3 and (E) nuclear NF-κB p65 levels determined by western blot analysis. HR resulted in lower p-Axl and SOCS3 levels and a higher NF-κB p65 level. Pretreatment with the Axl inhibitor R428 reversed the effects of Gas6 on p-Axl, SOCS3 and NF-κB p65. Data are expressed as the means ± SD (n = 4 per group). T-Axl: total Axl. p-Axl: phosphorylated Axl. HR: hypoxia-reoxygenation. Gas6: recombinant Gas6 20 ng/ml. R428: R428 100 nM. All values are expressed as the means ± SD (n = 4 per group). * P < 0.05 compared with the control group; *** P < 0.001 compared with the control group; ## P < 0.01 compared with the HR group.

We performed immunoblotting and quantitative real-time PCR (using TaqMan assays) to demonstrate the effect of HR on Axl expression in MLE-12 cells. Total Axl (T-Axl) expression was significantly decreased in HR group in comparison to the control group (Fig 7A). In addition, a significant decrease of Axl mRNA expression was found in HR group in comparison to the control group (Fig 7B).

To assess the role of Axl in the effects of Gas6, MLE-12 cells were pretreated with the Axl inhibitor R428 and with Gas6 before HR. We found that HR resulted in lower phosphorylated Axl (p-Axl) and SOCS3 levels and a higher NF-κB p65 level. Pretreatment with the Axl inhibitor R428 reversed the effects of Gas6 on p-Axl, SOCS3 and NF-κB p65 (Fig 7C–7E). These results indicated that Gas6 modulates the SOCS3-mediated pathway and attenuates HR-induced epithelial inflammation via up-regulating p-Axl.

### Gas6 up-regulates phosphorylated Axl in IR-ALI

**Fig 8:**
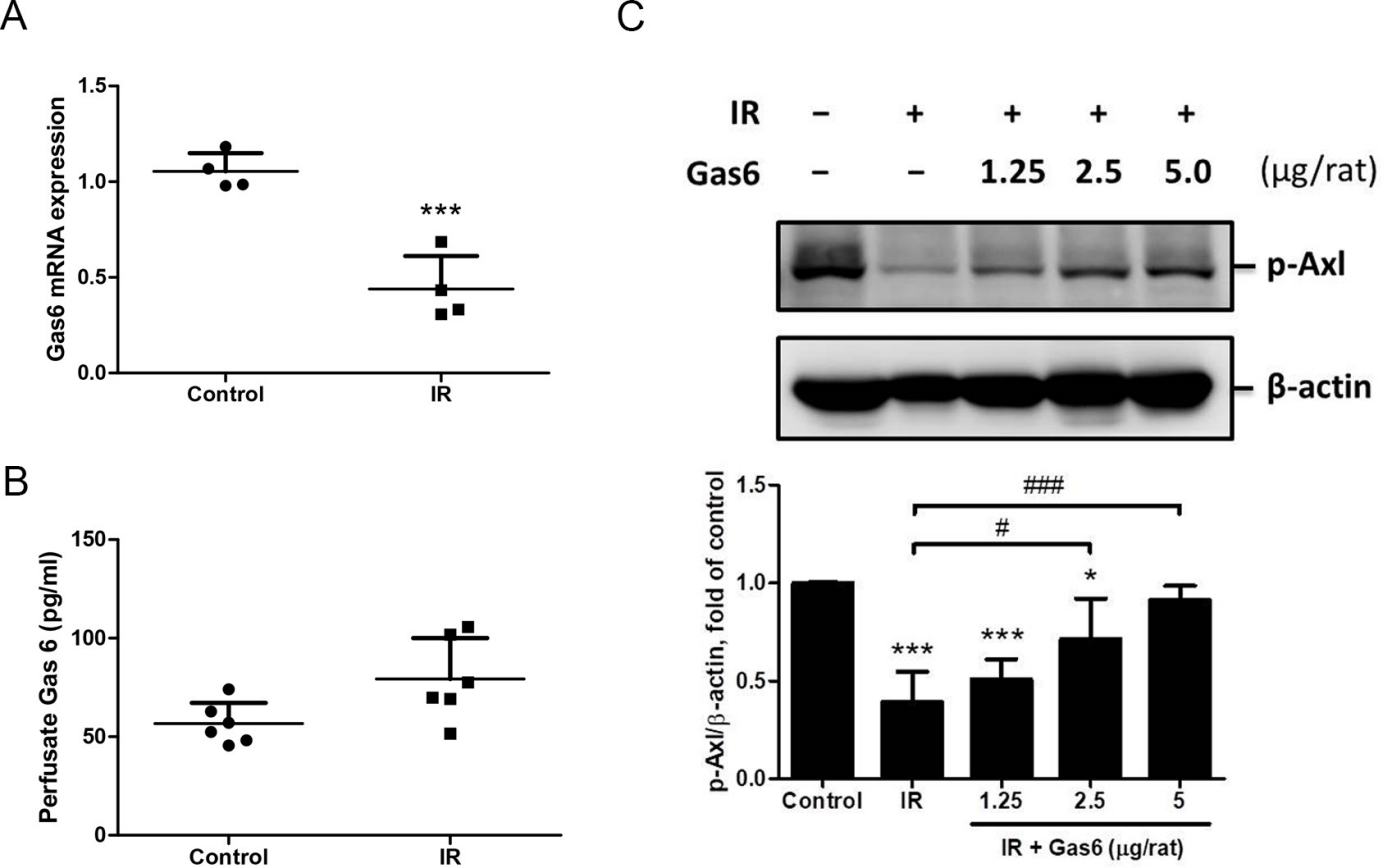
Gas6 and p-Axl of lung tissues. (A) Gas6 mRNA expressions in lung tissues. (B) Endogenous Gas6 levels in perfusate determined by ELISA analysis. (C) p-Axl levels determined by western blot analysis. Recombinant Gas6, but not endogenous Gas6, up-regulated phosphorylation of Axl in IR-ALI. p-Axl: phosphorylated Axl. IR: ischemia–reperfusion. Data are expressed as the mean ± SD (n = 4 per group for tissue mRNA and n = 6 per group for perfusate). * P < 0.05 compared with the control group; ** P < 0.01 compared with the control group; *** P < 0.001 compared with the control group; # P < 0.05 compared with the IR group; ### P < 0.001 compared with the IR group.

To demonstrate the effect of IR on endogenous Gas6 expression of rat lung, we performed quantitative real-time PCR (using TaqMan assays) for Gas6. A significant decrease of Gas6 mRNA expression was found in IR group in comparison to the control group (Fig 8A). We also measured the level of perfusate Gas6 by ELISA analysis. Compared with those in the control group, IR didn’t increase the perfusate level of Gas6 significantly (Fig 8B). To confirm the role of recombinant Gas6 in modulating the p-Axl expression ex vivo, the expression of p-Axl in rat lung with IR-ALI was measured. We found that IR resulted in lower p-Axl expression, which was restored by recombinant Gas6 with a dose-dependent manner (Fig 8C).

## Discussion

We have presented the first research regarding Gas6/Axl signaling in IR-ALI both ex vivo and in vitro. Gas6 protected the lungs against IR-related lung edema, inflammation, and damage. The anti-inflammatory effect of Gas6 on the alveolar epithelium was mediated by phosphorylation of Axl, which suppressed the activation of TRAF6 and NF-κB by up-regulating SOCS3-mediated pathway.

The role of Gas6 in the IR-induced injury of other organs has been studied previously. Gas6 suppressed the NF-κB and promoted cell proliferation, leading to the reduction of inflammation and protection of renal injury induced by IR [30]. In liver injury induced by IR, Gas6 acted as a survival factor for hepatocytes and reduced the production of inflammatory cytokines [31]. The present study demonstrated that Gas6/Axl signaling may attenuate the severity of IR-ALI, which supports the potential use of Gas6 in preventing IR-related organ dysfunctions.

It would be important to know if IR induced endogenous Gas6 in the system studied. We found tissue Gas6 mRNA level was decreased in IR-ALI, and IR didn’t increase the level of perfusate Gas6 significantly. Therefore, endogenous Gas6 induced by IR had limited effect in the present study. A previous study for Gas6 in hepatic IR had found that Gas6 mRNA levels of liver fell early after reperfusion. The increase in the level of endogenous Gas6 was time-dependent, and a significant increase of Gas6 level was detected by serum at 3 hours after reperfusion [31]. In the present study, variables of ALI were measured at 90 mins after reperfusion. We propose that increase level of endogenous Gas6 may emerge as a potential target to reduce ALI later, just as the findings in hepatic IR.

We found that Gas6 suppressed the production of IR induced proinflammatory cytokines in perfusates, such as CINC-1, IL-1β, IL-6, and TNF-α. Most previous studies about Gas6 for inflammatory diseases focused on its suppressive role in the innate immune system, including dendritic cells, macrophages, and neutrophils [16, 23, 27]. In the present study, the protection by Gas6 in the IR model may be partly attributed to its effects on alveolar macrophages and circulating leukocytes. However, the TNF-α level in BALF may represent the local generation of TNF-α from alveolar epithelium at the end of reperfusion. We found that Gas6 attenuated the increase induced by IR. The HR model represented the condition of Gas6 acting in the absence of disturbance from innate immune cells. Although Axl expression of alveolar epithelium was suppressed by HR, Gas6 up-regulated phosphorylation of Axl. The Gas6/Axl signaling attenuated HR-related inflammation of the alveolar epithelium. In ex vivo study, Gas6 also activated p-Axl specifically. These findings highlight that Gas6 directly modulates the alveolar inflammation via up-regulating phosphorylation of Axl in IR-ALI.

Gas6/Axl signaling reduced IR-ALI by up-regulating SOCS3 expression of alveolar epithelium in this study. Similarly, a previous study found that Gas6 attenuated inflammation via SOCS1 and SOCS3 in innate immune cells that encountered LPS stimulation [16]. The expression of SOCS3 is induced by various stimuli, including IR. SOCS3 binds to both Janus kinase (JAK) and cytokine receptors, which results in the inhibition of signal transducer and activator of transcription 3 (STAT3) [22]. Reduced SOCS3 expression has been found in rats with intestinal IR injury and mice with hepatic IR [32, 33]. Activation of the SOCS3/STAT3 signal transduction pathway might exert neuroprotective effects in rats with global cerebral IR injury [34].

The effects of SOCS3 in airway diseases have been studied previously. In these studies, the suppression of SOCS3 resulted in tissue damage, whereas its overexpression reduced disease progression [35–39]. The inhibition of SOCS3 mRNA expression is related to the imbalance of cytokine signaling observed in the bronchial epithelium of patients with chronic obstructive airway disease [39]. By up-regulating epithelial SOCS3, the inhibition of ROS production ameliorates lung inflammation induced by influenza A viruses [35]. In the models of ALI induced by high glucose and IgG immune complexes, SOCS3 overexpression is demonstrated to be a negative regulator of the JAK/STAT3 signaling pathway that reduces the inflammation of alveolar epithelial cells [36, 37]. To our knowledge, this is the first time that Gas6/Axl signaling has been associated with the SOCS3 expression of the alveolar epithelium in ALI.

We found that Gas6/Axl signaling suppressed the IR-induced activation of NF-κB. The TLRs are activated by a wide array of signals from tissues in response to oxidative stress, which initiates the inflammatory responses and the development of IR-ALI [8, 9, 40]. TRAF6 is an adaptor protein that participates in the transduction of upstream signals from TLR4, activates the downstream pathway of NF-κB, and leads to the production of pro-inflammatory cytokines [41–44]. SOCS proteins are intracellular immune regulators that modulate TLR signaling cascades [22, 45]. A previous study has found that the activation of SOCS1 inhibits the TLR4-TRAF6 signaling pathway in rat intestinal I/R injury [46]. In the present study, SOCS3 might have participated in the modulation of the TRAF6/NF-κB pathway by Gas6/Axl signaling in IR-ALI.

There are two main limitations in this study. First, the other TAMs, Tyro3 and MerTK, may have some immunomodulatory properties. Although Gas6 has the highest affinity for Axl receptor, and the effect of Gas6 is mediated by activating p-Axl specifically, the effects of these signals on IR-ALI were not measured in this study. Second, IR-ALI is a complex pathogenic condition that involves inflammatory cells, the pulmonary vascular endothelium, and the alveolar epithelium. The role of the endothelium of pulmonary vessels was not explored in this study. In other models of endothelial cells, Gas6 can abrogate apoptosis induced by injuries such as serum starvation, hypertonicity, and laminar shear stress [47–49]. In contrast, in mouse models of endotoxinemia, vasculitis, and heart transplantation, Gas6 promotes leukocyte extravasation, inflammation, and thrombosis by enhancing interactions between endothelial cells, platelets, and leukocytes [50]. Although the present study demonstrated the benefits of epithelial Gas6/Axl signaling in IR-ALI, further studies are warranted to identify the role of Gas6 in the crosstalk between the epithelium, endothelium, and inflammatory cells.

In conclusion, Gas6 up-regulates phosphorylation of Axl on alveolar epithelium in IR-ALI. The Gas6/Axl signaling activates the SOCS3-mediated pathway and attenuates IR-related inflammation and injury. Clinical studies are warranted to determine the therapeutic effects of Gas6/Axl signaling in IR-ALI in humans.

## Supporting information

S1 Checklist NC3Rs ARRIVE guidelines checklist. (PDF)

S1 Fig Raw images of western blot data. (PDF)

S1 Table Data of the effects of Gas6 on lung edema. (XLSX)

S2 Table Data of the expression of proinflammatory cytokines. (XLSX)

S3 Table Data of neutrophil count and lung injury score. (XLSX)

S4 Table Data of TNF-α and IL-6 in BALF. (XLSX)

S5 Table Data of endogenous Gas6 levels in perfusate and SOCS3, Axl, Gas6 mRNA expressions in lung tissues and MLE12. (XLSX)
