# Supplementary material for: Gas6/Axl signaling attenuates alveolar inflammation in ischemia-reperfusion-induced acute lung injury by up-regulating SOCS3-mediated pathway
Source: PLoS One. 2019 Jul 18;14(7):e0219788. doi: 10.1371/journal.pone.0219788 (PMC6638944; doi:10.1371/journal.pone.0219788)
Supplement: S1 Fig — (PDF) [file pone.0219788.s002.pdf]

S1_raw_images

Fig.4

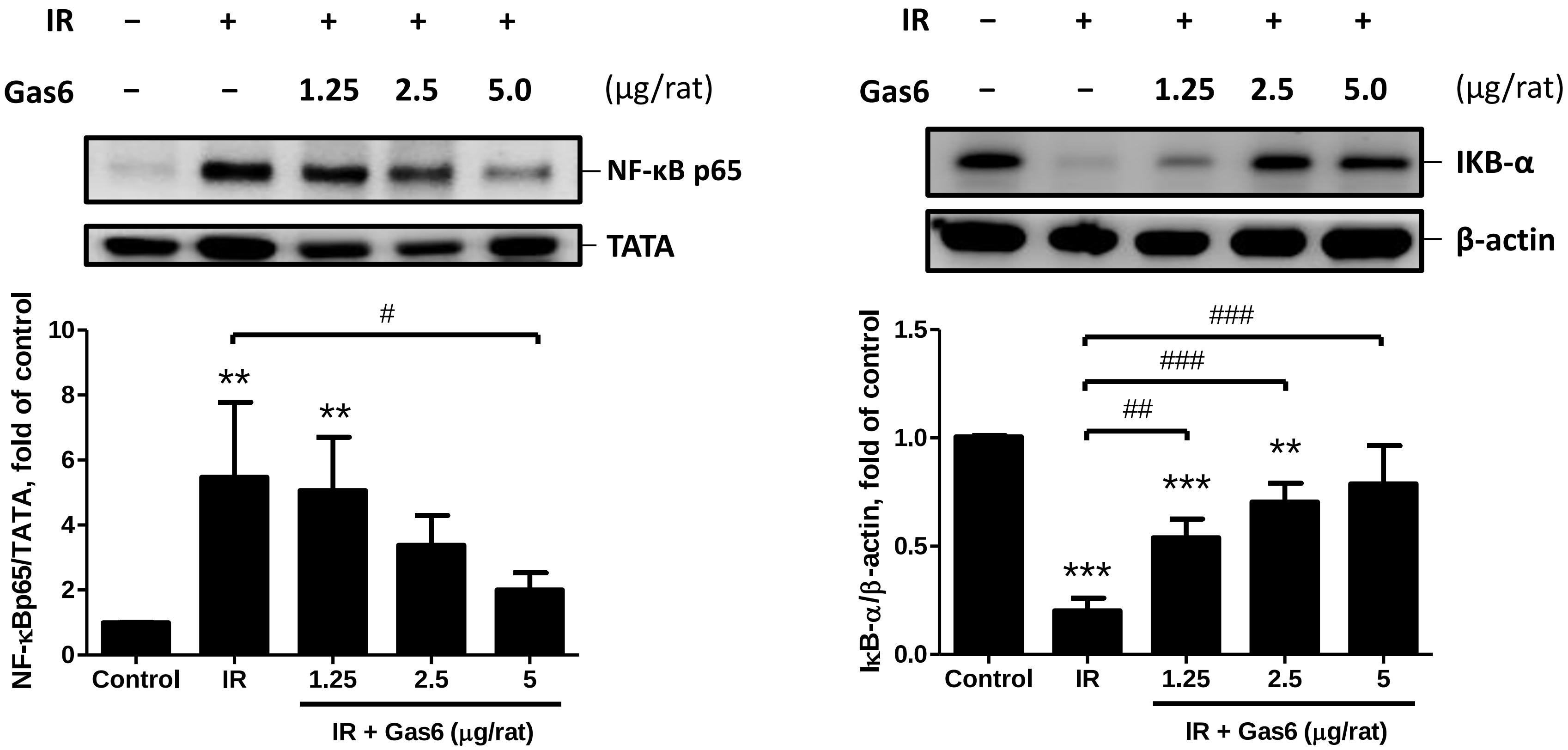

IR – + + + +
Gas6 – – 1.25 2.5 5.0 (μg/rat)
NF-κB p65
TATA
NF-κBp65/TATA, fold of control
0
2
4
6
8
10
#
**
**
Control
IR
1.25
2.5
5
IR + Gas6 (μg/rat)
IR – + + + +
Gas6 – – 1.25 2.5 5.0 (μg/rat)
IKB-α
β-actin
IκB-α/β-actin, fold of control
0.0
0.5
1.0
1.5
###
###
##
***
***
**
Control
IR
1.25
2.5
5
IR + Gas6 (μg/rat)

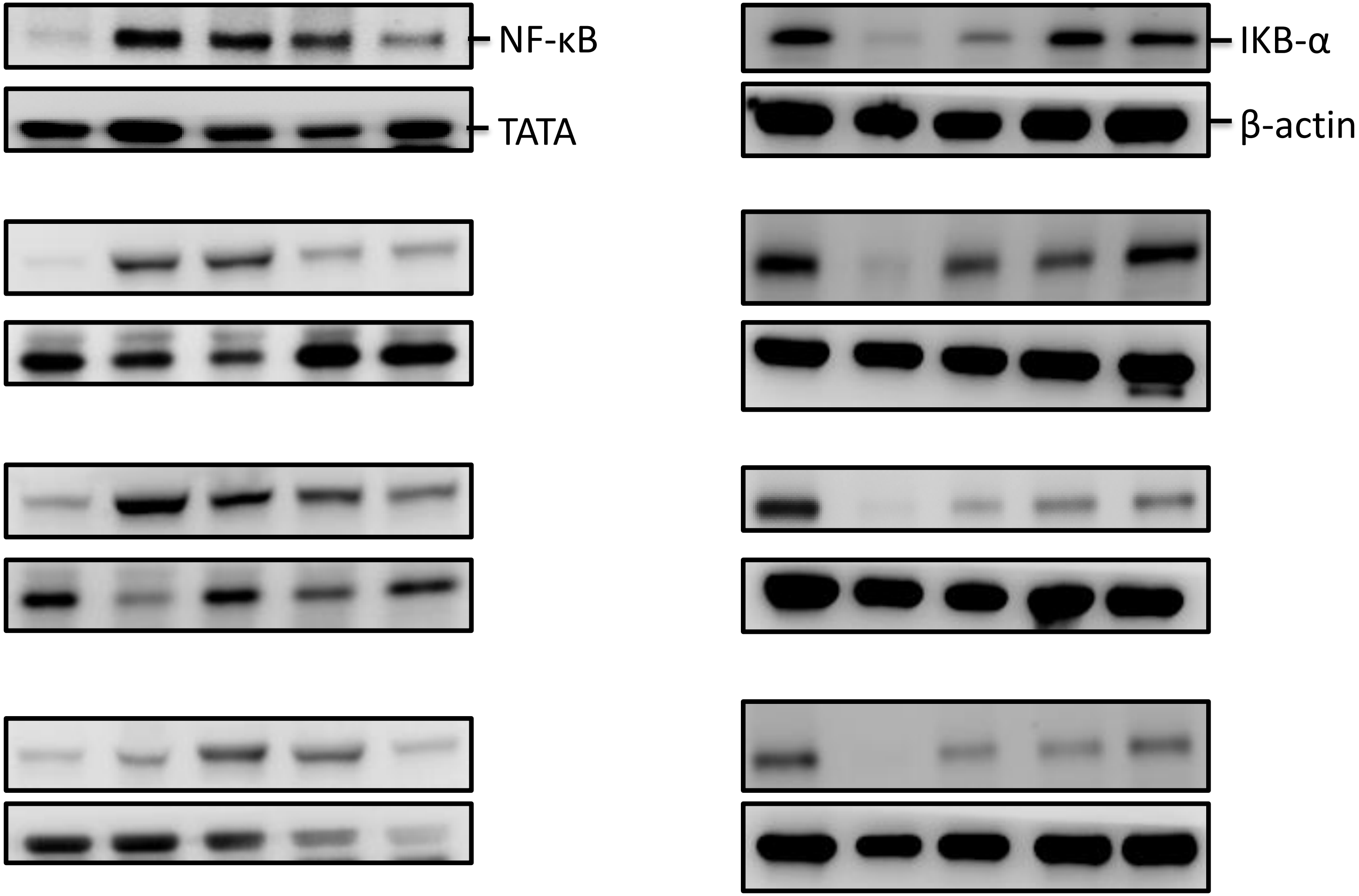
NF-κB
TATA
IKB-α
β-actin

Fig. 5 tissue SOCS3 WB

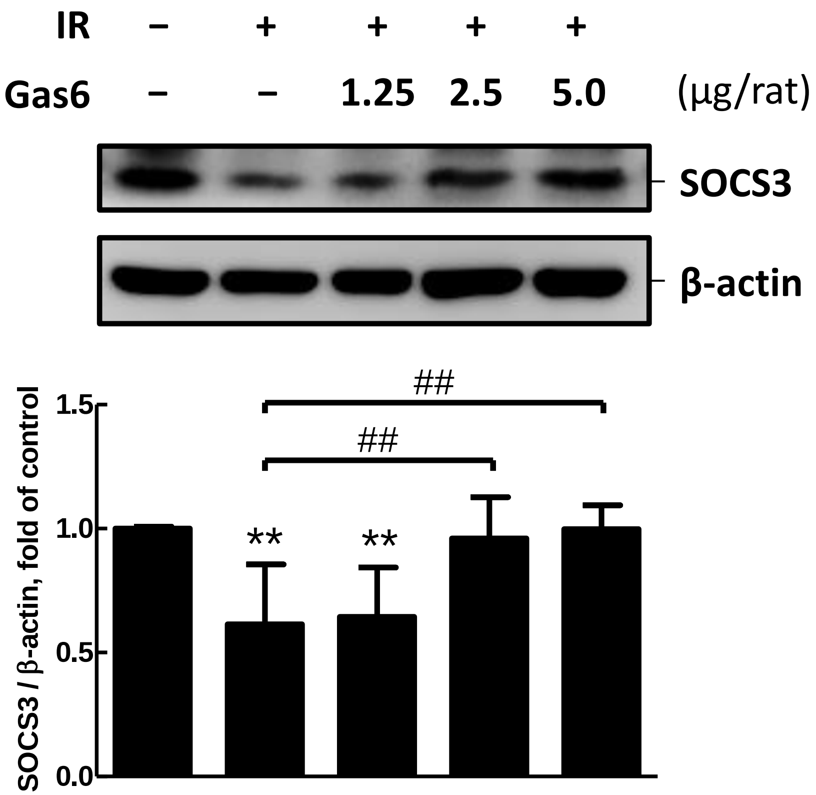

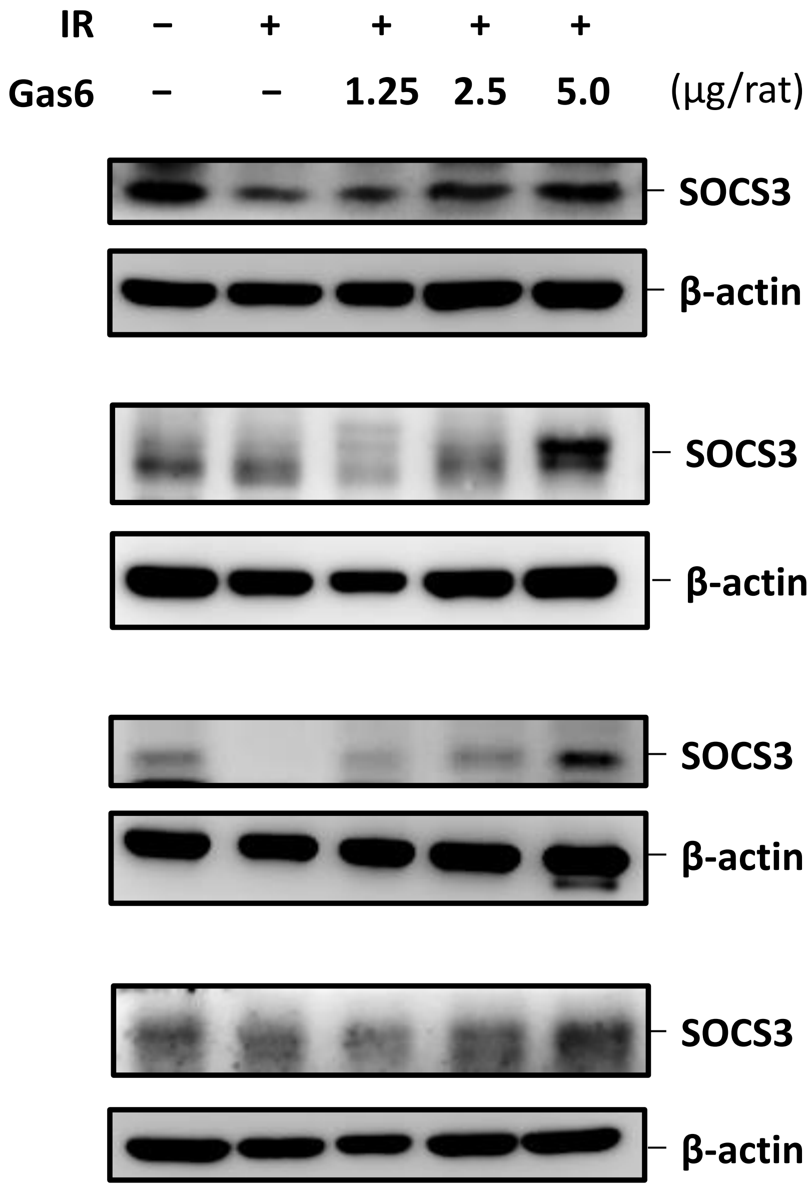
IR − + + + +
Gas6 − − 1.25 2.5 5.0 (μg/rat)
SOCS3
β-actin
SOCS3
β-actin
SOCS3
β-actin
SOCS3
β-actin

Fig. 6 cell dosage

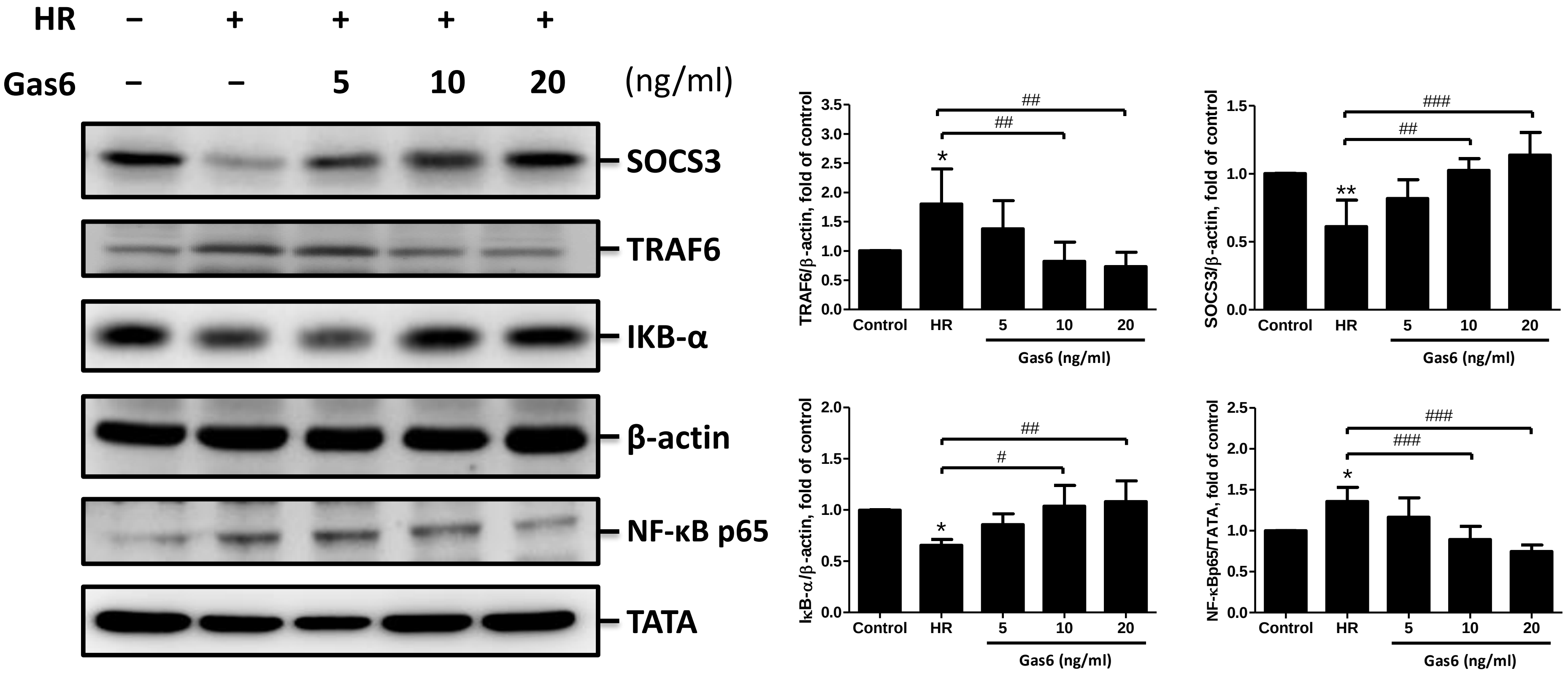

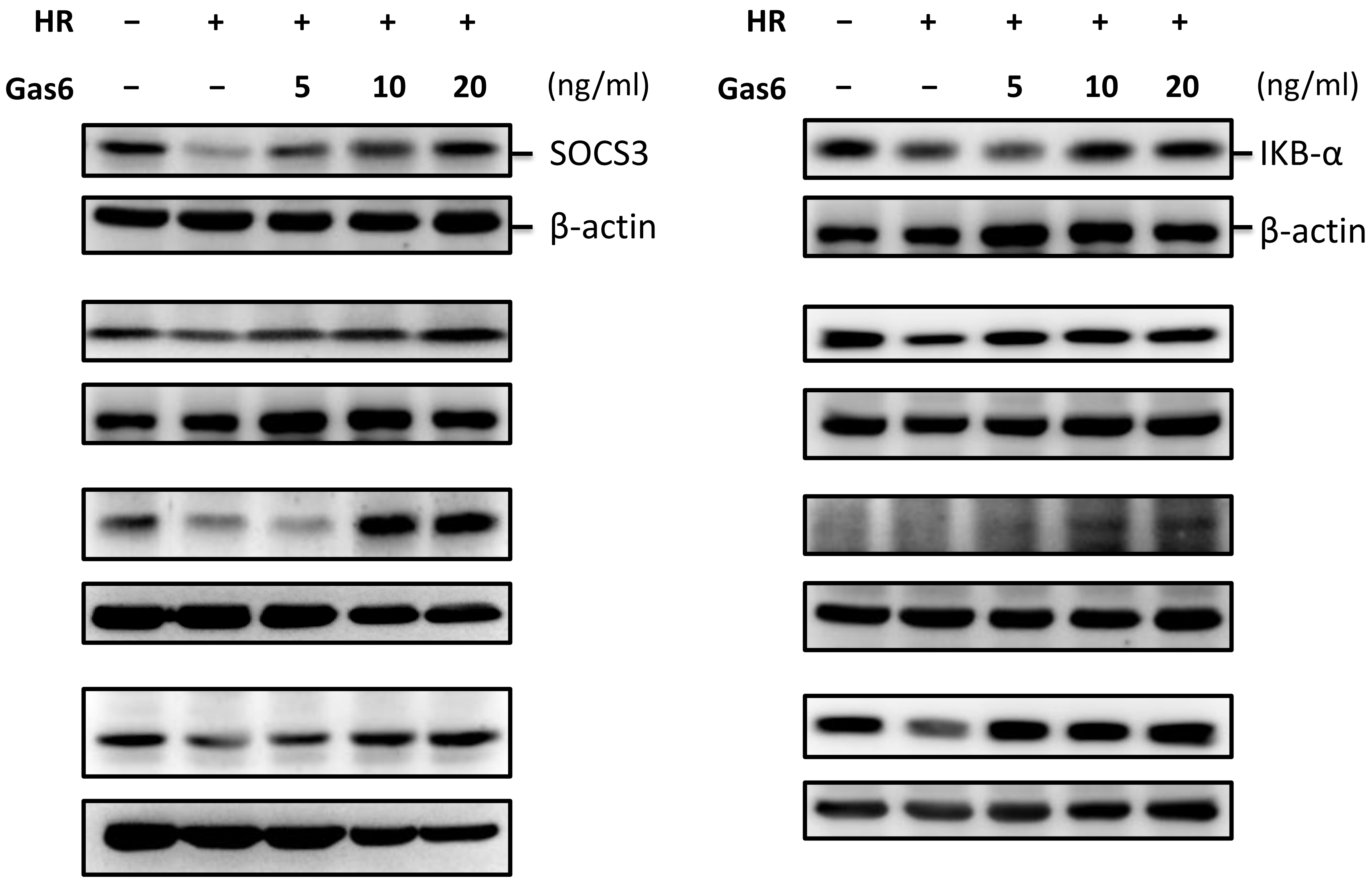

HR – + + + +
Gas6 – – 5 10 20 (ng/ml)
SOCS3
β-actin
HR – + + + +
Gas6 – – 5 10 20 (ng/ml)
IKB-α
β-actin

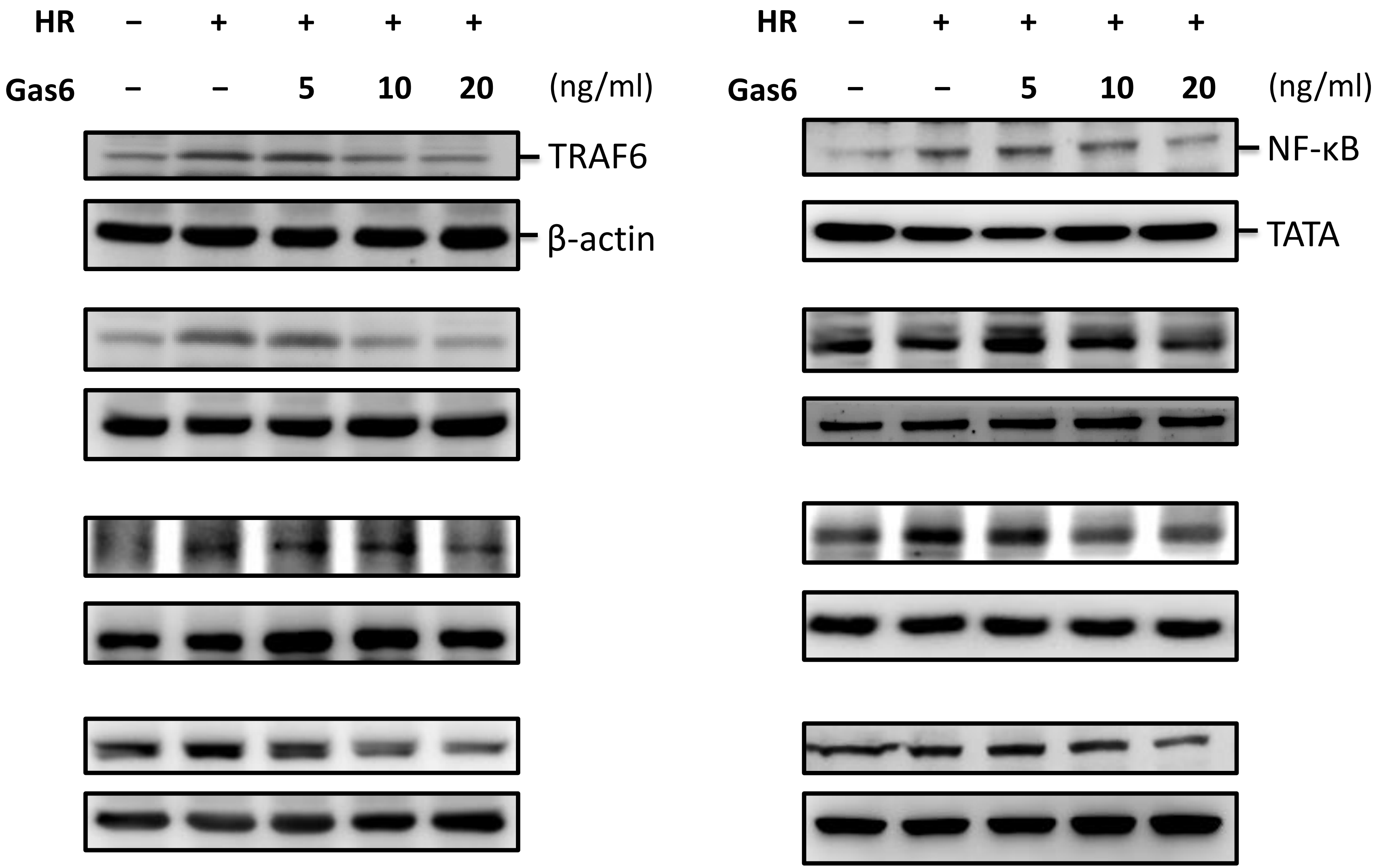
HR − + + + +
Gas6 − − 5 10 20 (ng/ml)
TRAF6
β-actin
HR − + + + +
Gas6 − − 5 10 20 (ng/ml)
NF-κB
TATA

Fig. 7 MLE12- HR +Gas6 +R428

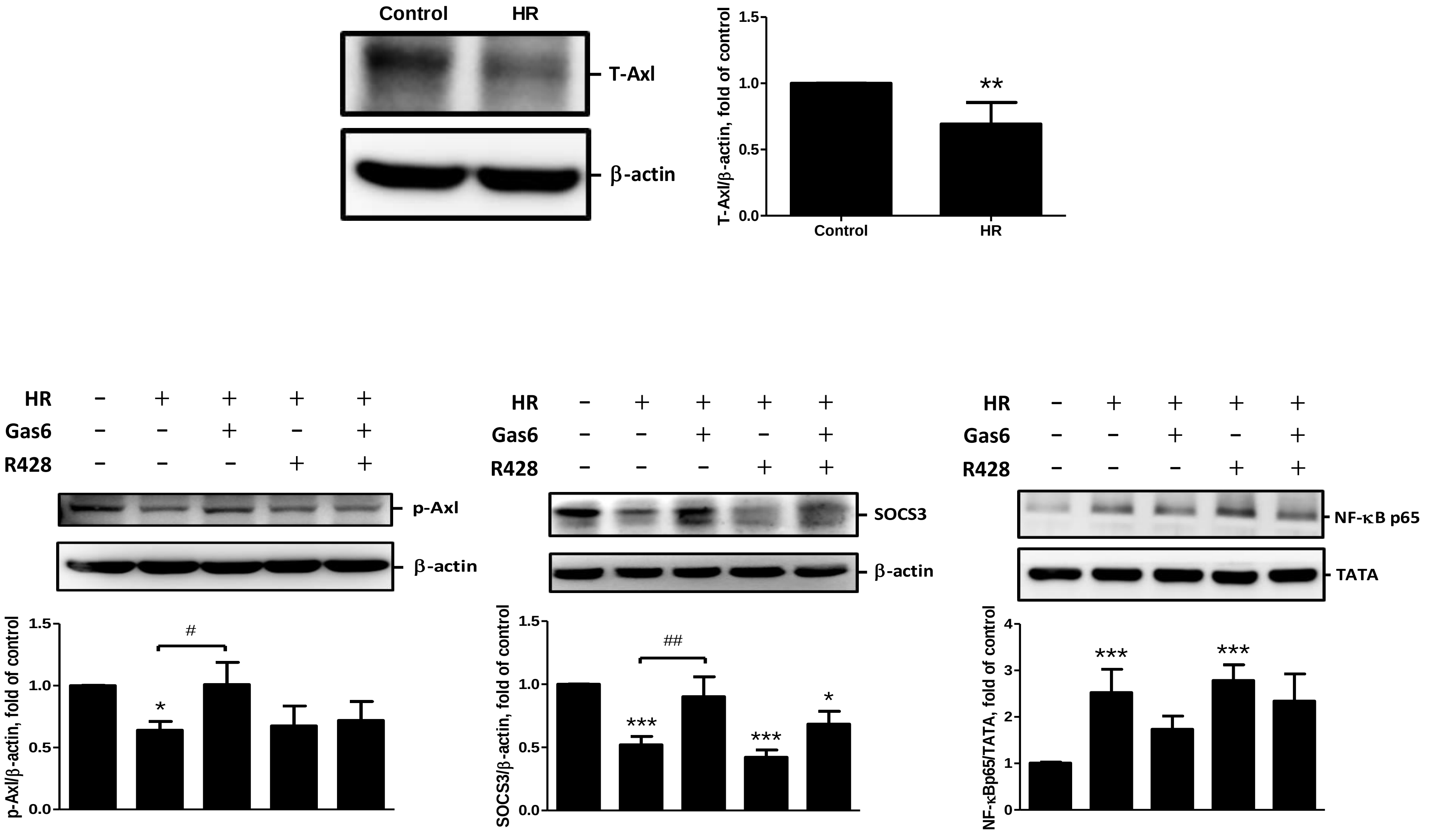

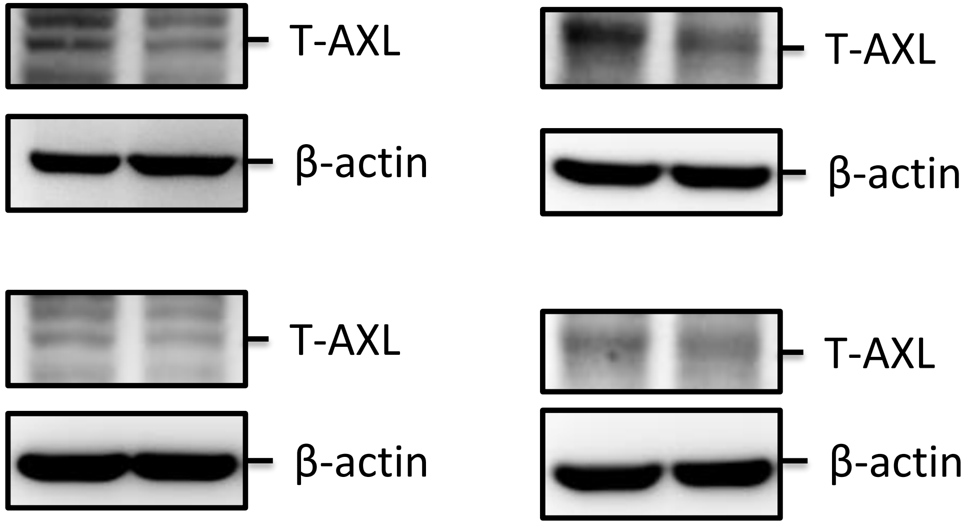
T-AXL
β-actin
T-AXL
β-actin
T-AXL
β-actin
T-AXL
β-actin

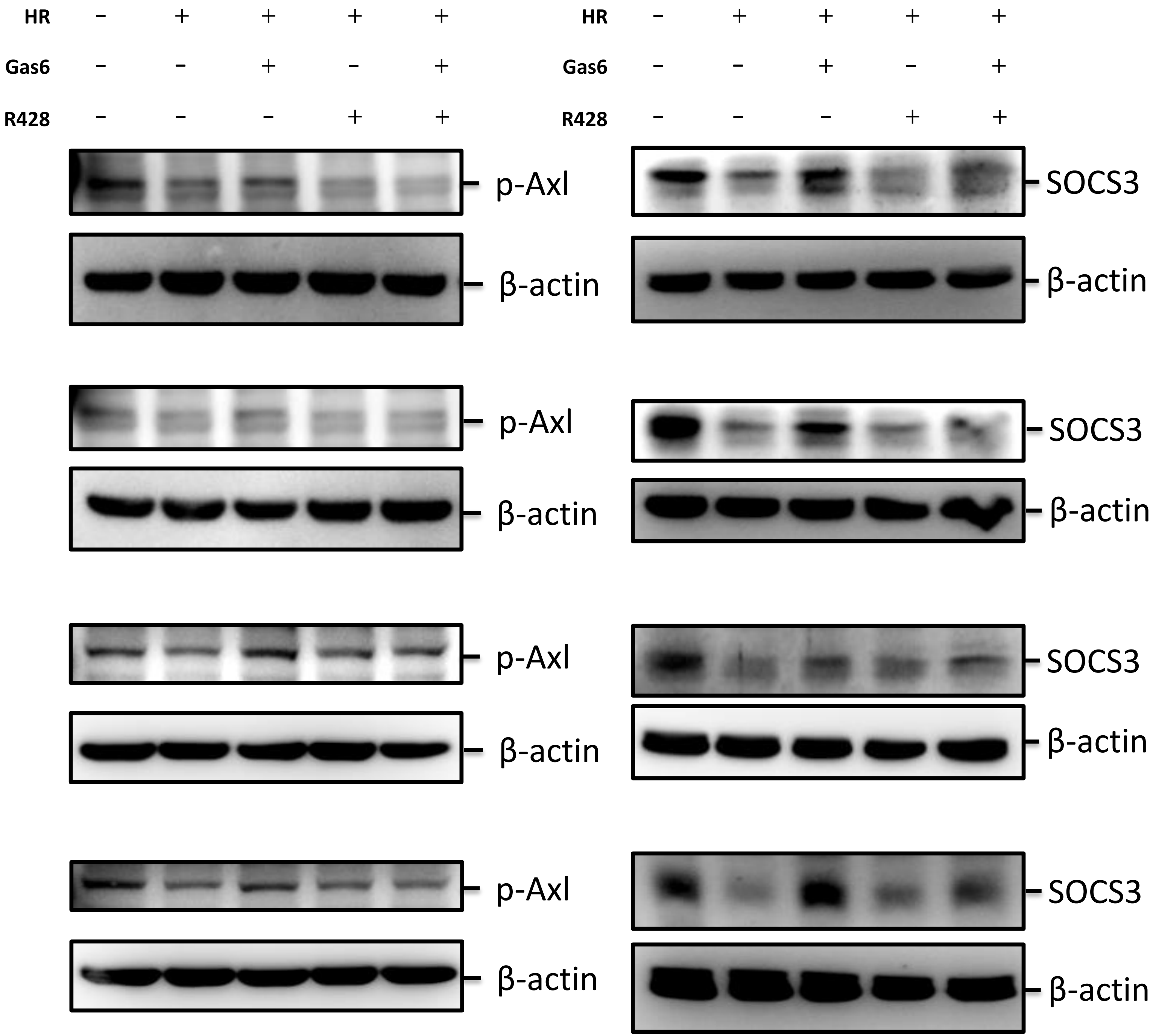
HR - + + + +
Gas6 - - + - +
R428 - - - + +
p-Axl
β-actin
p-Axl
β-actin
p-Axl
β-actin
p-Axl
β-actin
HR - + + + +
Gas6 - - + - +
R428 - - - + +
SOCS3
β-actin
SOCS3
β-actin
SOCS3
β-actin
SOCS3
β-actin

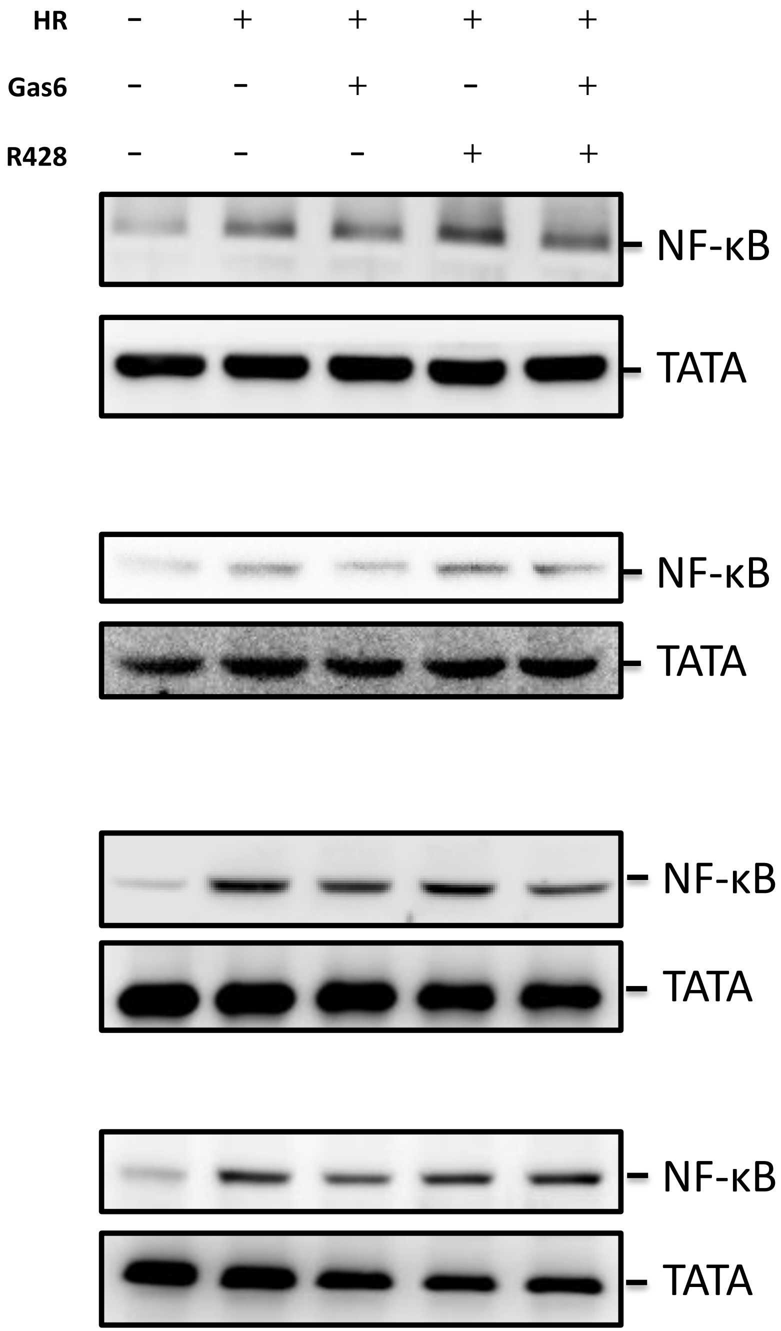
HR - + + + +
Gas6 - - + - +
R428 - - - + +
NF-κB
TATA
NF-κB
TATA
NF-κB
TATA
NF-κB
TATA

Fig. 8

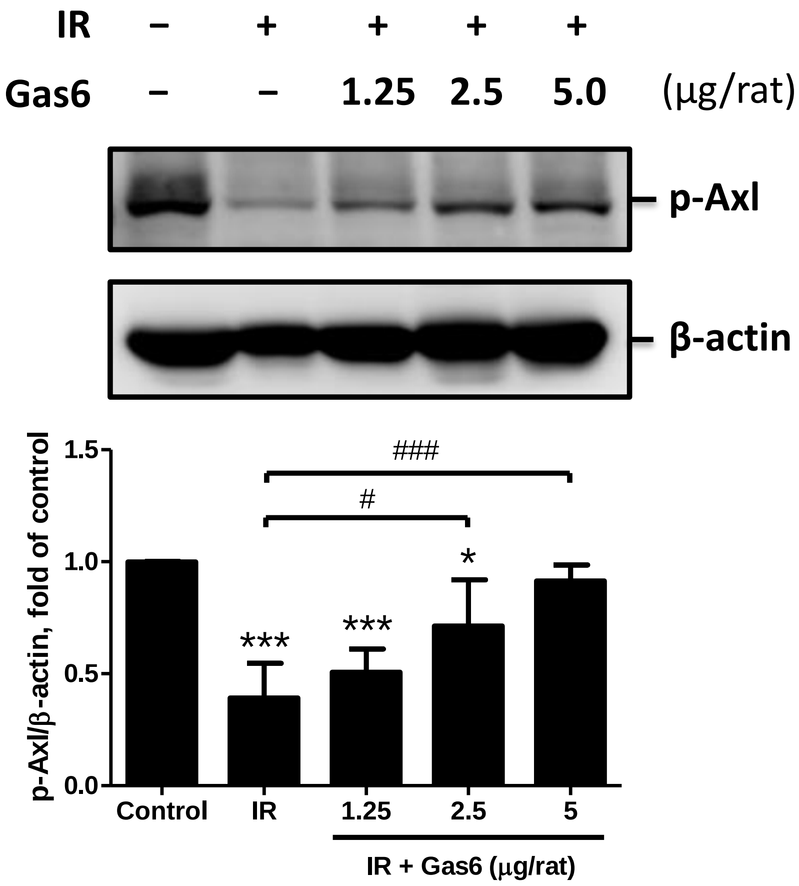
IR − + + + +
Gas6 − − 1.25 2.5 5.0 (μg/rat)
p-Axl
β-actin
p-Axl/β-actin, fold of control
1.5
1.0
0.5
0.0
###
#
*
***
***
Control
IR
1.25
2.5
5
IR + Gas6 (μg/rat)

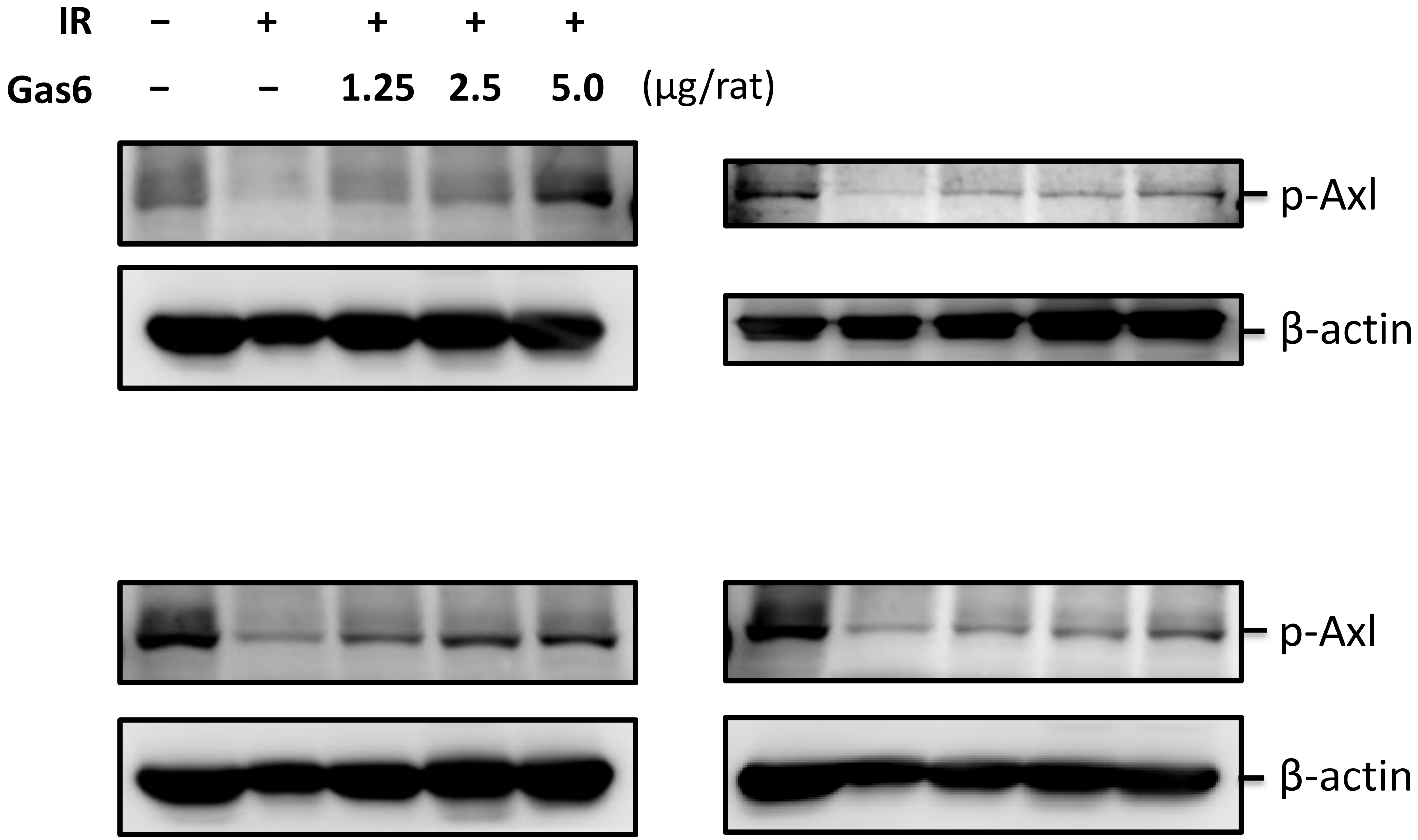

IR
−
+
+
+
+
Gas6
−
−
1.25
2.5
5.0
(μg/rat)
p-Axl
β-actin
p-Axl
β-actin
